# Prevalence, Patterns, and Predictors of Physical Inactivity in an Urban Population of India

**DOI:** 10.7759/cureus.26409

**Published:** 2022-06-28

**Authors:** Satyajit Mohanty, Jyotiranjan Sahoo, Venkatarao Epari, G Shankar Ganesh, Sandeep K Panigrahi

**Affiliations:** 1 Public Health, Institute of Medical Sciences and SUM Hospital, Siksha 'O' Anusandhan Deemed to be University, Bhubaneswar, IND; 2 Community Medicine, Institute of Medical Sciences and SUM Hospital, Siksha 'O' Anusandhan Deemed to be University, Bhubaneswar, IND; 3 Physiotherapy, Composite Regional Centre for Skill Development, Rehabilitation and Empowerment of Persons with Disabilities, Lucknow, IND

**Keywords:** sedentary behavior, behavior, epidemiology, social class, chronic disease

## Abstract

Physical inactivity (PI) is a risk factor for mortality and morbidity. PI and its predictors among the urban population in Bhubaneswar, India, were unknown. Finding out the contribution of PI as a cause of existing noncommunicable diseases (NCD) is difficult without following up with a cohort. The study was hence done to find out the prevalence, patterns, and predictors of physical inactivity in an urban population, and simultaneously investigate its causal relationship with NCD from this cross-sectional study. Cluster random sampling was used with a sample size of 1203 with a design effect of three. Socio-demographic, health profile, physical activity levels, and stage of change for physical activity behavior were collected. Logistic regression and marginal structural model analysis (by inverse probability of treatment weighting {IPTW} using a generalized estimating equation {GEE} to investigate the relationship between physical activity and prevalence of NCDs) were done using IBM SPSS v20 software (Armonk, NY: IBM Corp.). Statistical significance was tested at p=0.05. A total of 1221 subjects participated. The mean age was 35.25 years and 71.9% were physically inactive. General caste, presence of NCD, and being in a static stage of change influenced physical activity positively. PI was found to be a risk factor for NCD with 1.54 times higher odds in this population. The study concluded that the prevalence of physical activity was low and PI was a causative factor for NCD.

## Introduction

Physical activity (PA) is defined as any bodily movement produced by skeletal muscles that require energy expenditure, which includes structured activity programs such as exercises, and activities undertaken while working, playing, and carrying out household works [1]. Regular PA is known to improve physiological functioning, quality of life, and social and work participation. Considering the overall benefits of PA, the feasibility to engage and low cost of investment, the World Health Organization (WHO) has recommended a cumulative engagement of at least 150 minutes per week of moderate PA [2]. Despite the documented benefits and importance, 23% of males and 32% of females above 18 years of age are physically inactive around the world [2] and there is a global trend toward engaging in sedentary behaviors [3]. Compared to global estimates, Indians are found to be sedentary and less active physically. A multi-center study conducted in four regions of India concluded that more than 50% of the participants were inactive and less than 10% of the population engages in recreational PA [4]. Another study reported that the exercise intensities undertaken do not meet the global recommended standards, even in those who undertake leisure time PA [5].

Why few people are physically active and others are inactive is an intriguing question. Past exercise behaviors, perceived self-efficacy, social support, self-confidence, access to facilities, physical environment, gender, and socio-economic status are identified as some factors that can influence PA behavior [6]. Lower levels of motivation, limited free time, fear of falling, cost, transportation, pain, and lack of enjoyment are seen as barriers to participating in regular PA [7-9]. However, no previous work has specifically looked into the PA patterns and the factors that predict PA among the urban population in the Eastern part of India.

Further, noncommunicable diseases (NCD) account for 61% of total deaths in India and are attributed to lifestyle-related risk factors such as tobacco use, physical inactivity, alcohol consumption, and obesity [10,11]. Though promoting better urban design to increase PA and reducing sedentary lifestyles in urban India has been put forward as a strategy to reduce the risk of NCDs, we could not identify literature that has examined the relationship between PA and NCDs in India either as an independent risk factor or when controlled for other variables [12]. Therefore, we proposed to use inverse probability of treatment weighting (IPTW) method to investigate the relationship between PA and prevalence of NCDs in a large urban population.

This study aimed to find out the prevalence and patterns of physical activity in an urban population and to investigate the relationship of noncommunicable disease with physical inactivity.

The article was previously published as a pre-print: Mohanty S, Sahoo J, Venkatarao E, Ganesh Shankar G, Panigrahi SK: Prevalence, patterns and predictors of physical activity in urban population of Bhubaneswar smart city, India. MedRxiv. 2020. (DOI: 10.1101/2020.11.28.20240358).

## Materials and methods

This cross-sectional survey was conducted from February to August 2019 among adults of both sexes aged 18 years and above in Bhubaneswar, Odisha, India. Bhubaneshwar has a population of 8,85,363 residing in 1,97,661 households spread over 67 administrative wards and about 18.5% of the population reside in the slums as per Census 2011.

Sampling and sample size

The sample size was calculated based on previous estimates of the prevalence of PA (45.6%) in an urban setting [4]. Using a Z-value of 1.96 at 95% confidence, absolute allowable error at 5% with a design effect of three (to compensate for simple random sampling), the sample size was calculated to be 1203 to achieve 80% power.

Data collection tool and data collection

Thirty administrative wards were selected randomly and the households were selected using a systematic random sampling method. Door-to-door visits were conducted between 2 PM and 7 PM to collect the data. If a household refused to participate, the data were collected from the immediate next household. Only one response per household was documented. As the objective of the study was to document the prevailing PA patterns, individuals with physical and mental disabilities who could not undergo regular PA were excluded from the study.

A pre-tested, structured questionnaire was used to collect socio-demographic data - age, religion, caste, marital status, education, occupation, family size, and monthly family income. Respondents were asked if they were suffering from any previously physician-diagnosed chronic diseases such as hypertension, diabetes, cardiovascular disease, asthma, and other chronic respiratory problems. Questions pertaining to addiction to alcohol, and smoking were collected along with factors that promoted PA. The behavioral aspects related to stages of change to PA promotion were evaluated using Prochaska and DiClemente's model. The International Physical activity questionnaire (IPAQ) - short form was used to assess the PA level of the participants. The IPAQ- short form is a seven-day PA recall-based valid and reliable questionnaire and has seven questions related to frequency, duration, and intensity of PA. The average time taken to complete the questionnaire was about 15 minutes.

Classifications used

The socio-economic status (SES) of the participants was determined using a modified BG Prasad SES scale (2018) based on per capita monthly income. The SES was classified into five groups as per the modified BG Prasad classification system namely lower, upper-lower, middle, upper-middle, and upper class. Chronic diseases were classified as per the International Statistical Classification of Diseases and Related Health Problems, 10th revision (ICD-10). The state of change with respect to PA/exercise was grouped into either static (pre-contemplation and relapse) or dynamic (contemplation, decision, action, and maintenance) stages. Based on the IPAQ scores, PA levels were classified as low, moderate, and high. Participants who were classified as moderate PA and high PA were considered to be "physically active" for the purpose of this study. Respondents classified as having low PA was considered "physically inactive."

Statistical analysis

Statistical analyses were performed using the SPSS software v20.0 (Armonk, NY: IBM Corp.) licensed to the institute. Categorical variables were expressed in terms of frequency and percentages. Quantitative variables were expressed as mean ± standard deviation. Association between two categorical variables was tested using the Chi-squared test or regression analysis. Logistic regression analyses were used to compute adjusted odds ratios for each variable. a p-value of less than 0.05 was considered significant.

The causality of chronic disease by "physically inactive" was tested through inverse probability of treatment weighting (IPTW) by a marginal structural model (MSM) using a generalized estimating equation (GEE). Variables found to be associated with physical activity status in univariate analysis (with a p-value of less than 0.20) were used to generate the propensity score. First, the weighted predicted values (inverse propensity score) were calculated for PA categories, which were later normalized through a robust estimator for the covariance matrix applying generalized estimating equations for predicting the association of insufficiently physically active to chronic diseases.

## Results

A total of 1221 study participants were interviewed from the selected 30 clusters. After data cleaning, a total of 1125 cases were included for the analysis. The mean age of the study participants was 35.25 (± SD 10.72) years. Males constituted 55.9% of the participants. The average distance of the nearest public exercise facility from home was 496.81 (± SD 238.73) meters with a maximum distance of 1500 meters. Majority (87.4%) of the study population was in the dynamic stage of exercise behavior change according to the Prochaska and DiClemente model. A total of 809 (71.9%) respondents were classified as physically inactive. Rest 316 (28.1%) of the respondents were found to be physically active and among them, only 11 (1%) were involved in high PA.

Mean days/week spent in walking was 1.98 (95% CI: 1.80-1.16) with mean walk duration was 17.56 (95% CI: 16.76-18.37) mins/day. Mean days/week spend in "moderate intensity" PA was 0.33 (95% CI: 0.25-0.41) with mean "moderate intensity" PA duration (min)/day was 12.56 (95% CI: 11.88-13.25). Mean days/week spend in "high intensity" PA was 0.19 (95% CI: 0.13-0.25) with mean "high intensity" PA duration (min)/day was 11.44 (95% CI: 10.92-11.95).

Caste, educational attainment, chronic disease, and the stage of change were found to be associated with PA. Whereas, gender, religion, marital status, SES, employment status, family size, addiction, cumulative sitting duration, presence of neighborhood public exercise facility, or availability of neighborhood exercise facility within 500 meters do not have any association with PA (Table 1).

**Table 1:** Characteristics of the study population (n=1125) and their association with physical activity.

Variables	Total subjects (n, %)	Physically inactive (n, %)	Physically active (n, %)	Odds ratio (95% CI)	p-Value
Age					
18-29 years	379 (33.7)	265 (69.1)	114 (30.1)	0.89 (0.59-1.34)	0.593
30-39 years	324 (28.8)	247 (76.2)	77 (23.8)	0.64 (0.42-0.99)	0.047
40-49 years	271 (24.1)	195 (72.0)	76 (28.0)	0.52 (0.73-1.24)	0.342
50-59 years	151 (13.4)	102 (67.5)	49 (32.5)	Ref	-
Gender					
Male	683 (55.9)	423 (70.7)	175 (29.3)	1.13 (0.87-1.47)	0.350
Female	538 (44.1)	386 (73.2)	141 (26.8)	Ref	-
Religion					
Hindu	1053 (93.6)	758 (72.0)	295 (28.0)	0.94 (0.55-1.59)	0.833
Non-Hindu	72 (6.4)	51 (70.8)	21 (29.2)	Ref	-
Caste					
General	694 (61.7)	484 (69.7)	210 (30.3)	1.33 (1.01-1.74)	0.040
Others	431 (38.3)	325 (75.4)	106 (24.6)	Ref	-
Marital					
Unmarried	249 (22.1)	160 (64.3)	89 (35.7)	1.78 (0.63-5.02)	0.276
Married	855 (76.0)	633 (74.0)	222 (26.0)	1.12 (0.40-3.09)	0.824
Separated/widow(er)	21(1.9)	16 (76.2)	5 (23.8)	Ref	-
Education					
Above graduation	111 (9.9)	74 (66.7)	37 (33.3)	1.58 (1.02-2.44)	0.040
Up to graduation	423 (37.6)	286 (67.6)	137 (32.4)	1.51 (1.14-1.99)	0.003
Up to high school	591 (52.5)	449 (76.0)	142 (24.0)	Ref	-
Occupation					
Gainfully employed	575 (51.1)	424 (73.7)	151 (26.3)	1.48 (0.59-3.68)	0.395
Non-gainfully employed	519 (46.1)	360 (69.4)	149 (30.6)	1.84 (0.74-4.57)	0.189
Unemployed/retired	31 (2.80)	25 (80.6)	6 (19.4)	Ref	-
Family size					
Less than 5	905 (80.4)	661 (73.0)	244 (27.0)	0.75 (0.55-1.04)	0.088
More than equal to 5	220 (19.6)	148 (67.3)	72 (32.7)	Ref	-
Per capita					
Upper class	304 (27.0)	205 (67.4)	99 (32.6)	0.16 (0.01-1.56)	0.116
Upper middle class	491 (43.6)	361 (73.5)	130 (26.5)	0.12 (0.01-1.16)	0.067
Middle class	263 (23.4)	198 (75.3)	65 (24.7)	0.10 (0.01-1.07)	0.057
Lower middle class	63 (5.6)	44 (69.8)	19 (30.2)	0.14 (0.01-1.47)	0.104
Lower class	4 (0.4)	1 (25.0)	3 (75.0)	Ref	-
Chronic disease					
Yes	288 (25.6)	178 (61.8)	110 (38.2)	1.89 (1.42- 2.51)	<0.001
No	837 (74.4)	631 (75.4)	206 (24.6)	Ref	-
Addiction					
No	757 (67.3)	547 (72.3)	210 (27.7)	0.94 (0.72-1.25)	0.710
Yes	368 (32.7)	262 (71.2)	106 (28.8)	Ref	-
Availability of public exercise area					
Yes	995 (88.4)	716 (72.0)	279 (28.0)	0.97 (0.65-1.46)	0.920
No	130 (11.6)	93 (71.5)	37 (28.5)	Ref	-
Distance from the nearest exercise area (n=995)					
Up to 500 meters	726 (64.5)	522 (71.9)	204 (28.1)	1.01 (0.74- 1.38)	0.946
More than 500 meters	269 (23.9)	194 (72.1)	75 (27.9)	Ref	-
Behavioral stage (based on Prochaska scale)					
Dynamic	1046 (93.0)	801 (76.6)	245 (23.4)	0.03 (0.01- 0.07)	<0.001
Static	79 (7.0)	8 (10.1)	71(89.9)	Ref	-
Sitting duration/week					
<6 h/week	624 (55.5)	454 (72.8)	170 (27.2)	0.91 (0.70-1.18)	0. 481
>6 h/week	501 (44.5)	355 (70.9)	146 (29.1)	Ref	-

After adjusting for confounding factors, social caste, chronic disease status, and stage of change were found to be associated with PA. Caste and chronic disease status were the positive predictors whereas stage of change was found to be a negative predictor of PA in our study. General caste had 1.43 times higher odds of being physically active compared to reserved, which was statistically significant (95% CI: 1.03-1.99, p = 0.031). Respondents with chronic disease had higher odds of being physically active, i.e., 2.08 (95% CI: 1.46-2.98). Dynamic stage of change of exercise behavior had lower odds of being physically active, i.e., 0.02 (95% CI: 0.01-0.05).

A physically active person in Bhubaneswar was more likely from general caste, having some form of chronic disease and in the static stage of change. Age, education, employment status, family size, and SES did not exhibit any association with PA status (Table 2).

**Table 2:** Binomial logistic regression analysis of different factors associated with physical activity. SE: socio-economic

Variables	Adjusted odds ratio	95% CI	Beta	SE of beta	p-Value
Age					
18-29 years	0.71	0.42-1.21	-0.336	0.269	0.211
30-39 years	0.66	0.40-1.09	-0.412	0.257	0.108
40-49 years	0.87	0.54-1.42	-0.130	0.246	0.598
50-59 years	Ref	-	-	-	
Caste					
General	1.43	1.03-1.99	0.363	0.168	0.031
Reserved	Ref	-	-	-	-
Education					
Above graduation	1.30	0.71-2.37	0.266	0.306	0.385
Up to graduation	1.25	0.86-1.82	0.228	0.191	0.233
Up to high school	Ref	-	-	-	-
Employment status					
Gainfully employed	1.41	0.46-4.34	0.349	0.568	0.538
Non-gainfully employed	1.81	0.60-5.49	0.596	0.565	0.292
Unemployed/retired	Ref	-	-	-	-
Family size					
Less than 5	0.79	0.52-1.20	-0.228	0.212	0.282
More than equal to 5	Ref	-	-	-	-
SES					
Upper class	0.000	-	-18.651	22112.16	0.999
Upper middle	0.000	-	-18.739	22112.16	0.999
Middle	0.000	-	-18.729	22112.16	0.999
Lower middle class	0.000	-	-18.615	22112.16	0.999
Lower class	Ref	-	-	-	-
Chronic disease					
Yes	2.08	1.46-2.98	0.736	0.183	0.000
No	Ref	-	-	-	-
Prochaska scale					
Dynamic	0.02	0.01-0.05	-3.774	0.450	0.000
Static	Ref	-	-	-	-

Prior established determinants of the chronic disease were considered for our analysis, i.e., age, gender, behavior, marital status, education, occupation, neighborhood characteristics (presence of common exercise area), addiction, sitting time. Inverse probability of treatment weighting (IPTW) method and later generalized estimating equations (GEE) were used to explore the causal association between physical inactivity and chronic diseases based on the above-mentioned factors. Physically inactive subjects had higher odds (adjusted OR = 1.54) of having chronic disease (Table 3).

**Table 3:** Association of physical inactivity and chronic diseases (n=1125). SE: socio-economic

Variables	Adjusted odds ratio	95% CI	Beta	SE of beta	p-Value
Physically inactive	1.54	1.004 – 2.382	0.436	0.220	0.048

## Discussion

The study had a uniform representation of the study population of the urban city. There was no inclusion bias for gender as was seen during the analysis (p>0.05). The main results of the study show that the overall level of PA undertaken by the general public in the city is low. A mere 28.1% (316/1125) of participants were found to be physically active as measured by the IPAQ-short form. These scores were low compared to a work that reported 38.3% of the population to indulge in sufficient PA in India. Another international work that assessed PA from different countries found that only 9.3% of men and 15.2% of women are physically inactive in India. However, another study conducted in an urban city in South India reported a 49.7% prevalence of physical inactivity in a sample of 286 adults as measured by the WHO standard Global Physical Activity Questionnaire [13]. On the contrary, the results from a market intelligence agency showed that 64% of Indians in a sample of 3000 adults aged 18 years and above do not exercise [14]. This inconsistency in reporting PA prevalence patterns in India is perplexing. We hypothesize this to be attributed to the study population, location of study (urban/rural), type of questionnaire used to document PA or there may be rapidly growing physical inactivity in the urban Odisha.

Another alarming result that we found from our study was the 25.6% prevalence rate of participants suffering from at least a chronic disease. A recent study estimated the overall prevalence of diabetes, hypertension, and obesity in India as 2.9%, 14.4%, and 9.7%, respectively [15]. The results of our study showed that persons who suffer from chronic disorders are at increased odds of engaging in a regular PA program. Either we hypothesize to the prescription or advice by HCPs to participate in PA or the participants might have started doing exercises understanding the benefits of PA. This is contrary to previous results which found that the activity limitations increase with the number of chronic conditions and reduce the amount of PA undertaken in people with chronic disorders [16,17].

A total of 368 (32.7%) participants self-reported being addicted to illicit substances that are identified as risk factors that contribute to non-communicable diseases. This number (32.7%) is higher than the combined risk factors (alcohol use and tobacco use) provided in the 2014 report on the global status of non-communicable diseases [18]. The literature consistently reports that PA is inversely associated with tobacco use in adults and physically active adults are more likely to be moderate drinkers [19,20].

Our study results show that while education can influence PA patterns individually, the role of education seems to be silent when controlled for other sociodemographic factors. We hypothesize this to the role of perceived self-control executed by education on behavioral change. The study limitations such as having more than 50% of study participants who were educated till graduation level, PA was self-reported and was not reporting any long-term status change, the period of data collection was immediately before coronavirus disease (COVID)-pandemic with not so good national and global economic conditions might have played a role in the study period between predictors.

The results also showed that being in the dynamic stage of the Prochaska stage of change was found to negatively predict PA. The odds of those designated to be in a static stage of change (in Prochaska scale) are more likely to be actually physically active (according to IPAQ). Past studies have shown such changes to be more effective in participants who are already assigned to an intervention, or in those with pre-existing health disorders who have been assigned to an intervention [21,22]. IPAQ is more about what is practically done in the physical activity front, whereas the Prochaska scale is about intentions about self-perception of own’s physical activity. The Prochaska scale indicates the stage of change of behavior in relation to physical activity that includes what the respondent thinks about his/her physical activity status and what is his/her intentions about his/her physical activity. It has six designated stages. Accordingly, there are six different statements from which the respondent has to choose one that matches best for him/her. The stages of Prochaska are not permanent tags. People keep on changing through the stages. People who are designated as static have ample chances to be physically active and proceed to the dynamic stage. In our study as we have used IPAQ and Prochaska scale. In our case, we found many respondents in our study who were captured as physically active as per IPAQ but they thought that they are not physically active as per Prochaska. This was an interesting finding which was never reported before where the individual is actually physically active but he/she perceives he/she is not sufficiently so.

The result that physically inactive participants have higher odds of having a chronic disease was not surprising. PA is touted as a primary preventive measure for chronic disorders and fosters wellness in general [23]. PA also prevents the progression of symptomatic diseases and delays their progression to disability, or death [23,24].

Our data also showed a reduction in the duration and intensity of walking as measured by our study participants. The overall walking percentage of humans has reduced by 50-70% and a mean cadence of 7473 steps has been reported from 26 studies conducted between 1966 and 2007 [25,26]. Normative data indicates that normal adults walk between 4000 and 18,000 steps/day, and 10,000 steps/day is considered a reasonable cadence per day. Literature suggests that efforts have to be initiated to increase the walking to a minimum of 2000-2500 steps/day [27].

Among other factors studied, multi-variate analysis indicated that the general caste had 1.43 times higher odds of being physically active, when controlled for confounding factors. Social caste is a marker of socio-economic status in India [28]. Populations other than the general caste category fall into reserved caste and they represent the underserved population. Past work has reported that underserved populations were less likely to participate in sufficient moderate to vigorous PA [29].

There are some limitations to this study. For assessment of PA, IPAQ- short form was used, which is a self-reporting measure. The risks of recall bias leading to over or under-reporting of PA cannot be ruled out and future works may consider using objective measures of PA, such as pedometers or accelerometers. Further, IPAQ is criticized for overestimating PA prevalence in population surveys [30]. Future studies may evaluate the associations between PA prevalence measured by IPAQ, Global Physical Activity Questionnaire (GPAQ), and objective measures. Though a representative population has been sampled, the results may be generalized to other urban places but not relevant to rural areas. Considering the differences in PA prevalence between the results of this study and other previous works, efforts should be made to study the national estimate using a common protocol.

## Conclusions

Prevalence of physical activity was found to be 28.1% as per the short form of IPAQ questionnaire, out of which only 1% were involved in high level of physical activity. General caste was found to be a positive predictor of physical activity. Similarly, already having a chronic disease was also a positive predictor. People who are designated as static have ample of chances to be physically active and proceed to the dynamic stage. The odds of those designated to be in a static stage of change (in Prochaska scale) are more likely to be actually physically active (according to IPAQ). Physically inactive individuals were also found to have 1.54 times higher odds of having chronic disease than those who were physically active.
